# Profiling microorganisms in whole saliva of children with and without dental caries

**DOI:** 10.1002/cre2.206

**Published:** 2019-06-20

**Authors:** Alexandre R. Vieira, N. Luisa Hiller, Evan Powell, Leon Hak‐Jin Kim, Tracy Spirk, Adriana Modesto, Rachael Kreft

**Affiliations:** ^1^ Department of Oral Biology, School of Dental Medicine University of Pittsburgh Pittsburgh Pennsylvania; ^2^ Center of Excellence for Biofilm Research Allegheny Health Network Research Institute Pittsburgh Pennsylvania; ^3^ Department of Biological Sciences Carnegie Mellon University Pittsburgh Pennsylvania; ^4^ Department of Pediatric Dentistry, School of Dental Medicine University of Pittsburgh Pittsburgh Pennsylvania

**Keywords:** caries, IBIS Universal Biosensor, microbiota, *Streptococcus*, whole saliva

## Abstract

**Objectives:**

Dental caries is a highly prevalent infectious disease that causes tooth decay. While no single bacterial species is causative of dental caries, the role of the oral microbiome in oral health and caries is gaining interest. The purpose of this study is to compare the major species present in whole saliva samples from caries‐free and caries‐active children using the IBIS Universal Biosensor.

**Material and Methods:**

The abundant microbial species in ninety‐five whole saliva samples from caries‐free and caries‐active subjects were characterized using the IBIS Universal Biosensor.

**Results:**

Twenty‐four genera and sixty‐five species were detected. *Candida* and *Streptococcus* were common across samples, and often the dominant genus. While we did not observe a strong association between the most abundant species and oral health, *Bacteroides thetaiotaomicron* and *Rothia mucilaginosa* were enriched in children with active caries; while, *Staphylococcus epidermidis* was enriched in caries‐free children.

**Conclusions:**

These study trends observed suggest that microbial markers in saliva may serve as predictors of oral health and thus aid in diagnosis and treatments for prevention of caries. Consistent with competitive interactions, we also observed negative associations between *Streptococcus pneumoniae* and other streptococcal species, *Staphylococcus aureus* and *S. epidermidis, Candida* and *Neisseria*, and *Saccharomyces* and *Streptococcus*.

**Bullet Points**

- Role of the oral microbiome in oral health and caries.
- *Bacteroides thetaiotaomicron* and *Rothia mucilaginosa* enriched in children with active caries.
- *Staphylococcus epidermidis* enriched in caries‐free children.

## 1 INTRODUCTION

Dental caries is a highly prevalent infectious disease that causes tooth decay (National Institute of Dental and Craniofacial Research, 2018). In a 2010 survey of oral health status, untreated caries in permanent teeth and deciduous teeth rated as the first and tenth most prevalent conditions (Marcenes et al., 2013). The high prevalence of dental caries in humans is attributed to more frequent consumption of plant foods rich in fermentable carbohydrates in food‐producing societies. Comparable dental caries prevalence (higher than 50%) is documented for Pleistocene humans 15,000 years ago harvesting edible wild plants and for modern industrialized populations with a diet high in refined sugars and processed cereals (Humphrey et al., 2014).

Recent advances in sequencing technology reveal that the oral microbiota is highly diverse within a single individual (Human Microbiome Project Consortium, 2012). Within the oral cavity, some sites share communities, whereas others are highly distinct. Characterization of the oral cavity identified at least three communities: (a) the buccal mucosa, keratinized gingiva, and hard palate; (b) the saliva and tongue; and (c) the sub and supragingival plaques (Segata et al., 2012). In this ecologically rich environment, dozens of species, in multiple oral sites, can be associated with the pathogenesis of dental caries (Gross et al., 2012; Jiang, Zhang, & Chen, 2013; Yang et al., 2012). Although no single bacterial species is both a necessary and sufficient cause of dental caries, the role of the oral microbiome is oral health and caries is gaining increasing interest. Both the Ecological Plaque Hypothesis (1994) and the Keystone‐Pathogen Hypothesis (2012) suggest that oral disease is a consequence of microbial changes triggered by either environmental stress or bacterial‐induced inflammation (Hajishengallis, Darveau, & Curtis, 2012; Marsh, 1994; Rosier, De Jager, Zaura, & Krom, 2014). Thus, one of the important remaining questions regarding the etiology of caries is whether and/or how groups of strains, species, or genera shape the caries experience. Bacteria that are aciduric and acidogenic (such as *Streptococcus mutans* and *Lactobacillus* spp.) have been shown to promote caries and are frequently detected in dental carious lesions (Takahashi & Nyvad, 2011). However, these alone do not account for high frequency of caries (Gross et al., 2012). Instead, additional species, shifts in community structure, and overall diversity may also provide a pathogenic signature (Gomar‐Vercher, Cabrera‐Rubio, Mira, Montiel‐Company, & Almerich‐Silla, 2014; Gross et al., 2010; Yang et al., 2012).

In addition to bacteria, fungi also constitute an important component of the oral microbiome. The majority of microbiome studies uses 16SrRNA‐based technologies and thus do not account for eukaryotic microorganisms. The oral mycobiome is diverse in a healthy oral flora (Ghannoum et al., 2010), and some genera have been associated with caries. Studies in both rats and humans suggest *Candida albicans* may be a causal agent of caries (Klinke, Guggenheim, Klimm, & Thurnheer, 2011; Raja, Hannan, & Ali, 2010). Further, co‐aggregation between fungi and bacteria is also likely to influence the composition and metabolism of the oral microbiome, as exemplified by co‐aggregation between *C. albicans* and *S. mutans*. (Berbari et al., 2007; Jarosz, Deng, van der Mei, Crielaard, & Krom, 2009; Metwalli, Khan, Krom, & Jabra‐Rizk, 2013)

Studies of the oral microbiome have transitioned away from culture and into molecular detection, as an estimated 50% of oral bacterial species are unculturable. (Wilson, Weightman, & Wade, 1997) In this study, we employ the IBIS Universal Biosensor technology to capture abundant species in whole saliva samples. This technology makes use of polymerase chain reaction (PCR) to amplify microbial targets and mass spectroscopy (ESI‐MS) to detect the target DNAs (Ecker et al., 2008). The analysis does not require any prior knowledge of bacterial presence and is independent of culturing. The PCR‐MS identifies the species that contribute the most to the sample, and as such is ideal to classify the most abundant species; it is not optimized to capture component corresponding to 5% or less of the total DNA. Additional primers target *Staphylococcus* spp., leading to very high sensitivity to this genus. Another strength of the technique is that it detects *Candida* and *Saccharomyces*, using primers targeted at the 23S rRNA gene. There are multiple molecular technologies and relative strengths and weaknesses to each one. The PCR‐MS system (Universal Biosensor) is a powerful replacement to culture; it has been considered as an alternative to 16sRNA sequencing for routine diagnostics due to rapid turnaround time (from collection to results) and ability to distinguish DNA samples at the level of the species (Costerton et al., 2011; Lindsay et al., 2013). The largest comparison of molecular techniques for bacterial detection was performed on over 3,000 pediatric stool samples(Lindsay et al., 2013). The study contrasted the ability of the Universal Biosensor, 16‐s rRNA sequencing, and the GoldenGate Assay (Illumina) to detect seven enteric pathogens. The sensitivity of the Universal Biosensor ranged from highly sensitive to zero depending on the species. Overall, the Universal Biosensor and Golden Gate methods were the most similar methods.

In this study, we report the analysis of whole‐saliva samples from 95 subjects, including both caries‐free and caries‐active children to describe the major species present in the saliva and test potential correlations between abundant microbial species and disease state.

## 2 MATERIAL AND METHODS

### 2.1 Subjects

Frozen whole‐saliva samples from 95 children were used in this study; consecutive children being treated at the Pediatric Dentistry department were included. By the nature of the clinic, these children enjoy good overall health and have no issues that require dental care to be provided in a hospital setting. No children were excluded. Participants had not used antibiotics in the past month prior to biological sample collection. Children were asked to expectorate (spit) 1 ml of unstimulated saliva in a plastic vial. Saliva samples were collected at the beginning of the dental appointment, usually at 9:00 a.m. No subjects were recruited in the afternoon. Participants were at least 60 min without eating before sample collection. Saliva samples were immediately put on a container with ice and then stored at −80°C. Subjects were recruited at the Department of Pediatric Dentistry of the University of Pittsburgh, School of Dental Medicine between May and October 2012 as part of the Dental Registry and DNA Repository (DRDR) project. This study was approved by the University of Pittsburgh Institutional Review Board (approval number 0606091). All parents of the participating children provided written informed consent for their participation on the study after the children provided written assent for their participation.

### 2.2 Dental caries experience

Caries was diagnosed using the Decayed, Missing due to caries, Filled Teeth (DMFT) and Decayed, Missing due to caries, Filled tooth Surfaces (DMFS) scores. Teeth lost to trauma or primary teeth lost to exfoliation were not included in the final DMFT/dmft scores. When records indicated that teeth were extracted for orthodontic reasons, trauma or early periodontal disease or treatments were performed in sound teeth; these situations were not included in the final DMFT/dmft scores. Carious lesions were recorded as present when a break in enamel was apparent on visual inspection. All examinations were carried out under the supervision of an experienced specialist.

### 2.3 DNA extraction and IBIS Universal Biosensor BAC detection assay for microbial identification

For DNA extraction, the saliva was placed into a sterile microcentrifuge tube containing ATL Lysis buffer (Qiagen, Germantown, MD, cat# 19076) and proteinase K (Qiagen, cat# 19131). Samples were incubated at 56^°^C until lysis; 100 μl of a mixture containing 50 μl each of 0.1 and 0.7‐mm zirconia beads (Biospec cat# 11079101z and 11079107zx, respectively) were added to the samples, which were then homogenized for 10 min at 25 Hz using a Qiagen Tissuelyser. Nucleic acid from the lysed sample was then extracted using the Qiagen DNeasy Tissue kit (Qiagen cat# 69506). For microbiota analysis, 10 μl of each sample was loaded per well onto the Bacteria, Antibiotic Resistance, and Candida (BAC) detection PCR plate (Abbott Molecular, cat# PN 05 N13‐01). The BAC detection plate is a 96‐well plate, which contains 16 primers that survey all bacterial organisms by using the omnipresent loci (e.g., 16S rRNA sequences), whereas some are targeted to specific pathogens of interest (e.g., the Staphylococcus‐specific *tuf*B gene). The plate also includes primers for the detection of *Candida* species and some antibiotic resistance markers (e.g., mecA, vanA, vanB, and KPC). An internal calibrant of synthetic nucleic acid template is also included in each assay, controlling for false negatives (e.g., from PCR inhibitors) and enabling a semi‐quantitative analysis of the amount of template DNA present. PCR amplification was carried out, and the products were desalted in a 96‐well plate format and sequentially electrosprayed into a mass spectrometer. The spectral signals were processed to determine the masses of each of the PCR products present with sufficient accuracy that the base composition of each amplicon could be unambiguously deduced. Using combined base compositions from multiple PCRs, the identities of the pathogens and a semi‐quantitative determination of their relative concentrations in the starting sample were established by using a proprietary algorithm to interface with the IBIS database of known organisms.

### 2.4 Statistical analysis and visualization of Universal Biosensor data

Patients were organized based on DMFT scores of none (DMFT = 0) or high (DMFT ≥ 4 for children 6 to 12 and ≥7 for teenagers ages 13 to 19), these high cutoff values are 2 points above the average caries experience for each group. To display the distribution of species per patient, we generated heat map graphics using “image()” and “grid()” functions in “graphics” package part of R base distribution (version 3.2.2; R Foundation for Statistical Computing, Vienna, Austria). To calculate the strength of association between presence of single species and poor oral health (high DMFT), we employed relative risk, using a 95% confidence interval based on the standard error of the log relative risk (RR) ratio. To investigate positive (cooperative) or negative (competitive) relationships between bacterial species coexisting in same patient, we used one‐tailed Fisher's exact test in the R base distribution, and set up contingency tables with presence/absence of taxa 1 and presence/absence of taxa 2 (version 3.2.2; R Foundation for Statistical Computing, Vienna, Austria).

### 2.5 16S rRNA deep sequencing and analysis

To provide a general idea of how PCR‐MS compares with 16S rRNA sequencing in the oral microbiome of the whole saliva samples, we studied and selected three samples for deep sequencing by 16S rRNA gene using the Ion Torrent technology. The samples were selected because they displayed a range in the number of genera detected by PCR‐MS (from 1 to 4). Template preparation for emulsion PCR began with an initial PCR using "fusion" primers consisting of a 16S primer sequence, barcode adapter (GAT), Ion Xpress 10‐bp unique barcode, sequencing key (TCAG), and either Ion adapter A or trP1 (truncated P1). To allow bidirectional sequencing, two sets of primers were designed for each amplicon, one with adapter A and one with trP1 (Table S1). Hypervariable regions V3 and V6 from the 16S rRNA gene were selected for amplification. The PCR was performed with Invitrogen High‐Fidelity Platinum Taq Polymerase (Life Technologies cat. no. 11304‐011) using a ~1–5‐ng/μl template, 55°C annealing temperature, and 30‐s extension for 35 cycles.

The PCR products were purified by adding a 1.8× volume of Agencourt AMPure XP magnetic beads (Beckman Coulter cat. no. A63881) to each sample, washing with fresh 70% ethanol and eluting in 30ul of 1X TE. A 20 ul was recovered to avoid bead aspiration, and 1 ul of each was visualized using the Agilent 2100 Bioanalyzer with the DNA 1000 kit (cat. no. 5067‐1504). Using the resulting nmol/L quantities, all samples were pooled to a 5,000‐pM/ea concentration and subsequently serially diluted down to 25 pM**.** Ten different dilutions were run on an Agilent High‐Sensitivity chip (cat. no. 5067‐4626), and the dilution that contained the target amplicons closest to 26 pM was selected for the emPCR. The emPCR was done on the Ion OneTouch 2 instrument according to the Ion PGM™ Template OT2 400 Kit protocol (Pub. no. MAN0007218 v2.0), the amplified sample was enriched on the Ion ES system, and sequencing prep was done using the Ion PGM™ Sequencing 400 Kit (Pub. no. MAN0007242 v2.0) using an Ion 316 chip V2 on the PGM platform. The adapters were removed from the output reads, and fastq formatted files were downloaded for each sample. Read lengths longer than 100 bp were parsed out from each file, MIDs were removed, and sequences were submitted to the RDP Classifier 2.7. OTUs were assigned to the best hit, using the default confidence cutoff of 0.8. We reported all phyla identified and genera with a minimum of 100 reads (Tables S2 and S3).

## 3 RESULTS

### 3.1 Description of subjects and detection technology

The subject set was composed of 95 subjects recruited to the Dental Registry and DNA Repository. All subjects were organized by age and oral health (Table S4). Subjects ranged from 6 to 19 years of age, where 44 were female and 51 male. The DMFT and DMFS scores were used to assess oral health, where high scores describe poor oral health. The DMFT scores ranged from 0 to 17 and were divided into three subcategories according to caries experience level in children and teenagers (Table 1).

**Table 1:** Information on patient population

Median age	13
Percent male	53
Percent Caucasian	22
Percent African American	61
DMFT median	2
DMFT range	0–17

### 3.2 Overall microbial composition of whole saliva using PCR‐MS

**Figure 1:**
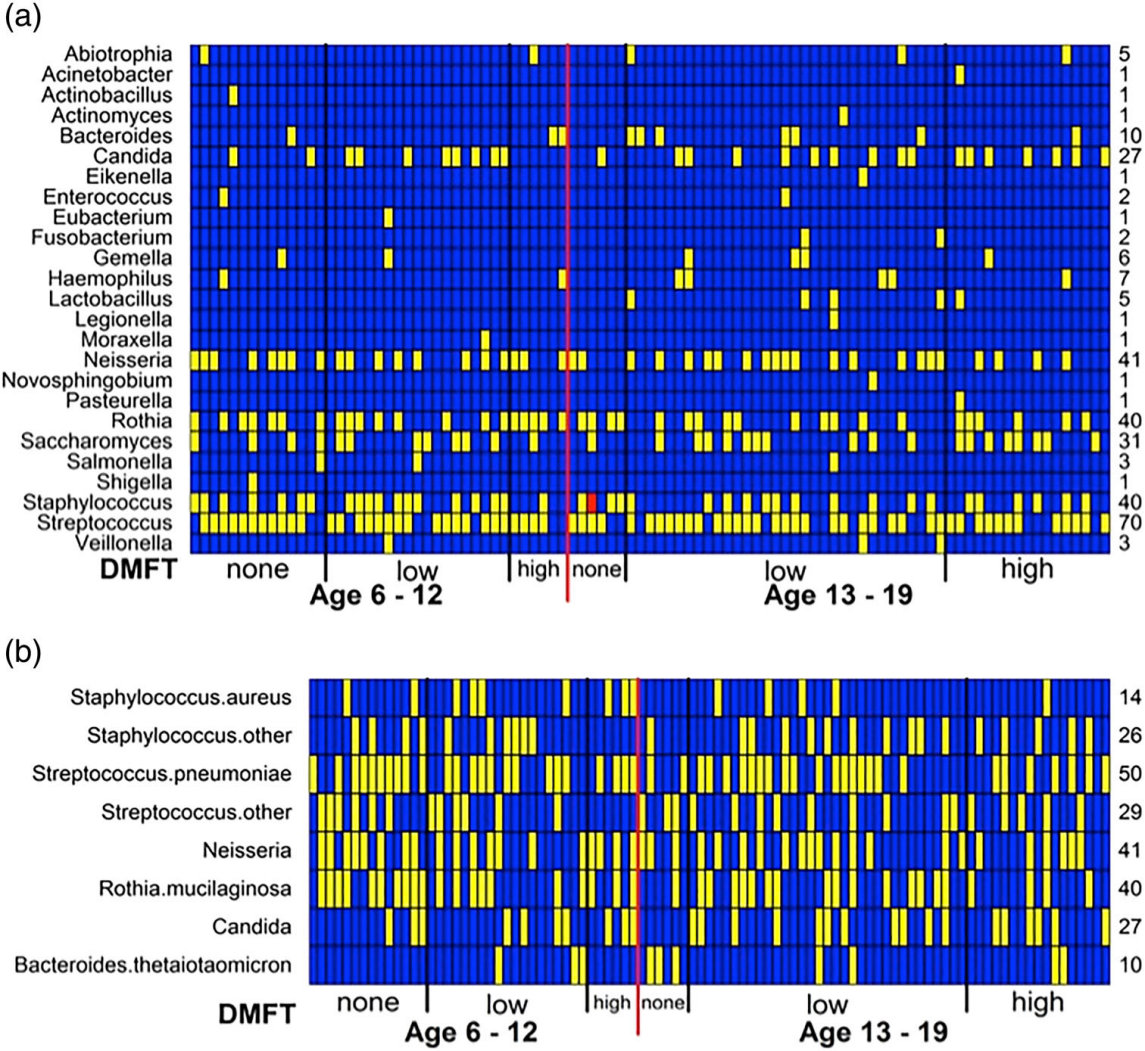
Characterization of the abundant species in whole saliva using PCR‐MS. (a) Genera detected by PCR‐MS. (b) Most common genera and species encountered in the 95 subjects. Columns represent subjects. Rows represent species or genus, where blue denotes absence, yellow presence, and red presence of bacteria and the mecA gene coding for methicillin resistance. Columns are sorted into two age groups, separated by a red line. Each group is sorted into three levels of caries experience, separated by black lines. Age 6–12 categories are none/DMFT = 0; low/DMFT = 1–3; high/DMFT = 4 or higher; and age 13–19: none/DMFT = 0; low/DMFT = 1**–**6; high/DMFT = 7 or higher. The numbers on the right margin are total number of subjects with the respective species or genus (note that some subjects have multiple species from a single genus)

Sixty‐five species, within 24 genera were identified by PCR‐MS within the 95 samples (Tables S4–S6). The most common genera detected were *Streptococcus*, *Neisseria, Staphylococcus*, *Rothia, Saccharomyces, and Candida* (Figure 1a and Table S5). The most common species encountered was *S. pneumoniae*, present in 53% of subjects (Figure 1b and Table S6); note the PCR‐MS characterization of this species can incorporate close streptococcal species such as *Streptococcus pseudopneumoniae or Streptococcus mitis*. Two *Staphylococcus* species were dominant, *S. epidermidis* (19%) and *S. aureus* (15%). Eighty‐three percent of the species was identified in five or less of the samples, suggesting extensive diversity in the major contributors to saliva composition across this pediatric population. Furthermore, in the majority of samples (81/95), we detected multiple species (range of 2–8 species/sample) consistent with the absence of a single dominant species. Average species detection was 3.75 unique bacteria or fungi per sample. In fourteen samples, we detected only one species (six were streptococci, and four were fungal) consistent with very high abundance in the saliva (Table S4).

### 3.3 Association between microbial genera and oral health status

We tested the hypothesis that high abundance of a subset of species may serve as markers of disease severity by searching for associations between species and disease severity. The samples were organized into two age groups (Group 1 ages 6–12 and Group 2 ages 13–19) and two disease categories (none or high caries). None was defined as DMFT = 0, that is, no caries. High was defined as DMFT ≥4 in children and DMFT ≥7 in teenagers (Table 2). RR analysis was used to identify species that when present could be associated with cavities; a RR > 1 implies cavities where more likely to occur in subjects where the species was present, and a RR < 1 implies cavities were less likely to occur in subjects where the species was present (Table 3 and Table S7). *Neisseira* and *Streptococcus* were commonly encountered genera; however, their detection was not associated with poor oral health. In contrast, detection of *B. thetaiotaomicron* and *R. mucilaginosa* were more common in young children with cavities. Furthermore, *S. epidermidis* was detected in children without cavities but not those with cavities suggesting it may serve as a marker of oral health in young children (Table 3). Finally, saliva samples contained streptococci and staphylococci and one single example of methicillin resistant *S. aureus*. The presence of these genera in samples suggests that the saliva may serve as a source of transmission for the upper respiratory microbiome.

**Table 2:** Description of oral health measurements and distribution over subject set

Caries experience level	Number of individuals
Children (from 6 to 12 years of age)	39
None: DMFT = 0	14
Low: DMFT = 1–3	19
High: DMFT = 4 or higher	6
Teenagers (from 13 to 19 years of age)	56
None: DMFT = 0	6
Low: DMFT = 1**–**6	33
High: DMFT = 7 or higher	17

**Table 3:** Analysis of the associations between species and oral health

	All						Child				
Species	DMFT	Present	Absent	RR	Lower CI	Higher CI	Present	Absent	RR	Lower CI	Higher CI
*Bacteroides thetaiotaomicron*	HighNone	3	20	1.463	0.769	2.782	2	4	2.833	0.877	9.151
		1	19				1	13			
*Candida*	HighNone	7	16	1.444	0.844	2.470	0	6	0.000	0.000	NA
		3	17				2	12			
*Rothia mucilaginosa*	HighNone	11	12	0.875	0.501	1.529	5	1	3.333	0.473	23.471
		11	9				7	7			
*Staphylococcus aureus*	HighNone	3	20	1.463	0.769	2.782	1	5	1.800	0.373	8.681
		1	19				1	13			
*Staphylococcus epidermidis*	HighNone	3	20	0.436	0.160	1.188	0	6	0.000	0.000	NA
		8	12				4	10			
*Streptococcus*	HighNone	16	7	0.885	0.492	1.592	4	2	0.667	0.171	2.604
		15	5				11	3			
*Streptococcus pneumoniae*	HighNone	11	12	0.875	0.501	1.529	2	4	0.500	0.117	2.139
		11	9				8	6			

*Note*. Relative risk (RR) was used to measure the relative risk of cavities being associate with presence of a single species. RR > 1 denotes higher risk of cavities with presence of species, whereas RR < 1 a lower risk of cavities with presence of species. The confidence intervals (CI) serve to estimate the true relative risk in the population (vs. the sample set). The confidence level was set at 95%. NA: non‐applicable due to very low numbers.

### 3.4 Association between species colonizing whole saliva

Bacterial species can cooperate or compete; thus, the species encountered in the whole saliva were analyzed for positive and negative associations. The species and genera present in four or more subjects were compared in pairwise combinations for association using Fisher's exact test. Both streptococci and staphylococci displayed evidence of intergenus competition. Specifically, *S. pneumoniae* displayed a negative association with other streptococcal species and *S. aureus* a negative association with both *S. epidermidis* and the set of all other staphylococcal species (Table 4). We also uncovered evidence for interkingdom competition where *Candida* was negatively associated with *Neisseria*, and *Saccharomyces* was negatively associated with *Streptococcus* (Table 4).

**Table 4:** Taxa displaying negative associations within whole saliva

Taxa 1	No	Taxa 2	No	Co‐occurrences	P value
*Streptococcus pneumoniae*	50	Streptococci excluding *S. pneumoniae*	29	9	0.005
*Staphylococcus aureus*	14	Staphylococcus excluding *S. aureus*	26	0	0.008
*Staphylococcus aureus*	14	*Staphylococcus epidermidis*	18	0	0.041
*Candida*	27	*Neisseria*	41	6	0.008
*Saccharomyces*	31	*Streptococcus*	70	18	0.017

Abbreviation: No: number of occurrences.

### 3.5 Deep sequencing of the 16S rRNA gene on three samples

**Figure 2:**
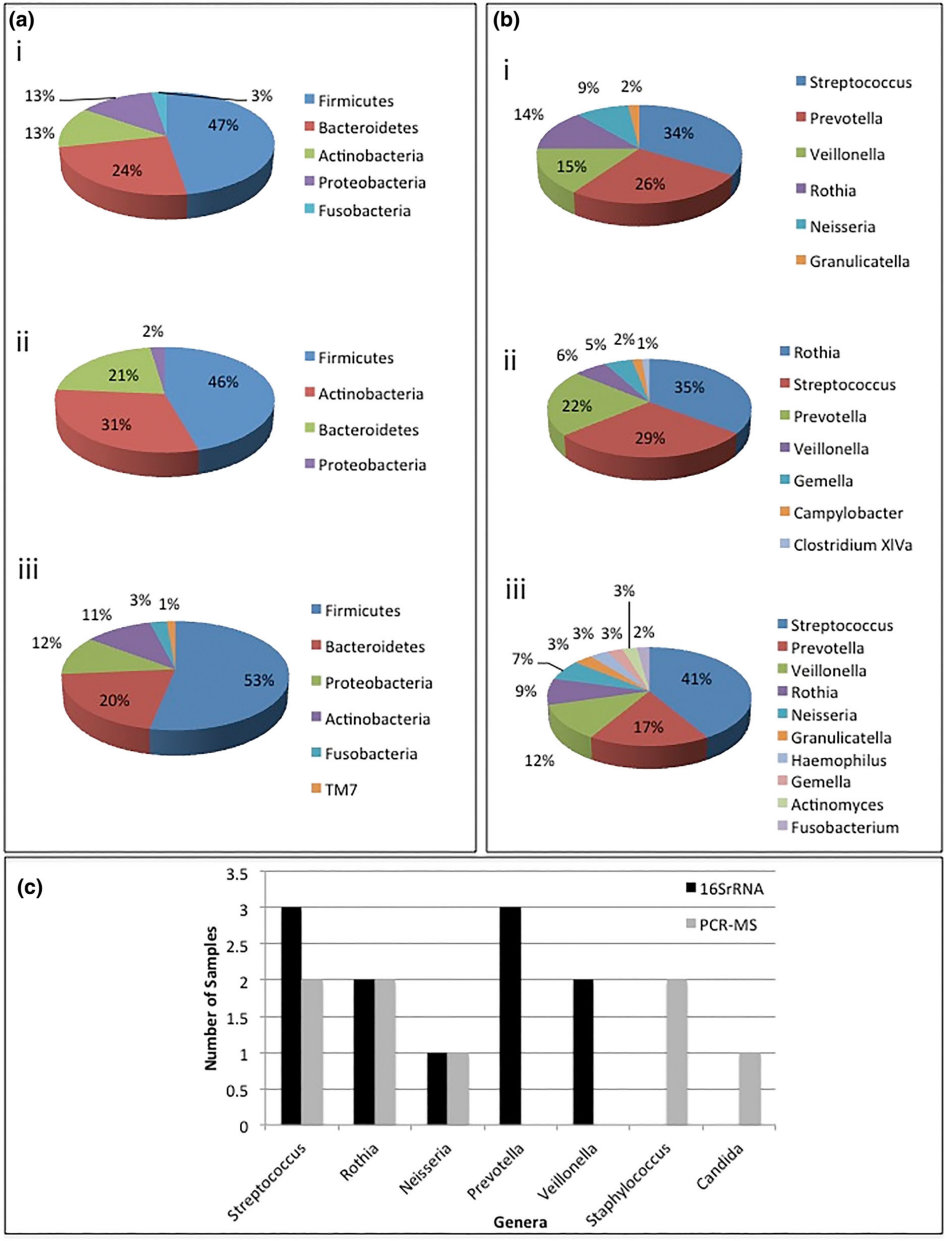
Comparison of PCR‐MS and 16S rRNA sequencing for three subjects. (a) Phyla identified by 16S rRNA sequencing. (b) Genera identified by 16S rRNA sequencing. (c) Comparison between PCR‐MS and 16S rRNA sequencing analysis for three samples

We compared microbiological profile between PCR‐MS and 16S rRNA sequencing for three samples (Figure 2a,b) to investigate the sensitivity of the PCR‐MS. PCR‐MS detected between four and six genera per sample whereas 16S sequencing detected between six and 10 genera. By PCR‐MS, the three samples displayed a range in the number of genera detected from 1 to 4. Both PCR‐MS and 16S rRNA sequencing detected a prevalence of *Streptococci*, *Neisseria*, and *Rothia* in the samples (Figure 2c). Sequencing of 16S rRNA gene suggests that *Prevotella* and *Veillonella* were not detected by PCR‐MS; whereas staphylococci was not detected by 16S rRNA sequencing.

## 4 DISCUSSION

This study employed PCR‐MS to characterize the most abundant microbial species in 95 saliva samples from caries‐free and caries‐active subjects. Twenty‐four genera with 65 species were detected. We observed trends between the presence of microbes and oral health. These must be evaluated in larger sample sets, where, if they hold true, would suggest that microbial markers in saliva may serve as predictors of oral health and thus aid in diagnosis and treatments for prevention of caries. Previous studies suggest that the microbial composition of saliva may serve to indicate systemic disease, such as pancreatic cancer and heart disease (Farrell et al., 2012). Finally, previous work has shown similar microbial composition in saliva, throat, and tonsils (R Core Team, 2018). In agreement, we detect upper respiratory colonizers, streptococci, and staphylococci, in saliva. The saliva may also serve as a marker for colonization of the upper respiratory tract.

The presence and importance of *S. aureus* in saliva is a topic of debate. In this study, we detected seven *Staphylococcus* species, the most prevalent were *S. epidemidis* and *S. aureus*. This finding is in agreement with other studies that have identified *Staphylococcus* spp. in the oral cavity of healthy subjects as well as those with periodontal disease (Martins CA de, Koga‐Ito, & Jorge, 2002; Ohara‐Nemoto, Haraga, Kimura, & Nemoto, 2008; Petti, Boss, Messano, Protano, & Polimeni, 2014; Smith et al., 2003). Specifically, in the analysis of saliva from healthy subjects, Ohara‐Nemoto and colleagues identified *Staphylococcus* in 83.9% of samples, and Petti and colleagues identified *S. aureus* in 43% of samples (Ohara‐Nemoto et al., 2008; Petti et al., 2014). *S. aureus* competes with multiple members of the microbiome, such as *S. epidermidis* (Lina et al., 2003; Otto, Echner, Voelter, & Götz, 2001). In this manner, its prevalence in the oral cavity may play an important role in shaping the oral microbiome. Given that *S. aureus* is a major community‐acquired pathogen, establishing its prevalence in the oral cavity is pivotal for a complete understanding of its reservoirs and transmission.

The major species identified in this study was *S. pneumoniae*. The genus *Streptococcus* has been shown to dominate the oral cavity; the most commonly detected species are *S. parasanguinis*, *S. salivarus*, and *S. infantis* (Human Microbiome Project Consortium, 2012). Similarly, *S. mutans* has been shown to be highly associated with caries (Takahashi & Nyvad, 2011). Unexpectedly, we did not detect *S. mutans*; this may reflect the specific population or a lack of detection by the Universal Biosensor. The prevalence of *S pneumoniae* in our sample set may reflect the patient population or biases of the PCR‐MS technology. Either way, it suggests that the saliva may play a role in the transmission of this important opportunistic pathogen (Levine et al., 2012).

Saliva samples serve as biomarkers of oral and systemic disease, but they do not correlate closely with the microbial composition of supragingival pockets or plaque. The microbiota from saliva is more diverse than that of plaque and yet more stable over time (Human Microbiome Project Consortium, 2012). The diverse community may prove to be very useful to analyze cooperative or competitive behaviors among taxa. Our studies suggest there may be competition between a few taxa pairs; prominent was the negative association within the genera *Streptococcus* and *Staphylococcus*. *Streptococcus* spp. and *Staphylococcus* spp. are known to encode for multiple bacteriocidal molecules that play a role in inter or intraspecies competition, all of which are candidates for competitive interaction in whole saliva (Dawid, Roche, & Weiser, 2007; Lina et al., 2003; Lux, Nuhn, Hakenbeck, & Reichmann, 2007; Maricic, Anderson, Opipari, Yu, & Dawid, 2016; Otto et al., 2001). However, these negative associations should be cautiously interpreted, given that the bacteria could still be present, even if not detected. In this manner, the relevance of these ecological associations must be investigated in larger sample sets with deeper coverage to establish whether they play a role in oral colonization and health. If accurate, such behaviors could be direct and/or indirect, mediated not only by surface or secreted microbial molecules but also via host antimicrobial and inflammatory responses.

In accordance with previous work done in whole saliva (Yang et al., 2012), we demonstrated that dental caries active disease status has a distinct microbiological profile in comparison with healthy mouths. Our findings provide a rationale for exploiting salivary microbiomes as diagnostic markers for dental caries disease status and possibly other conditions such as periodontal diseases. This type of analysis is very practical, as either a professional or a parent can perform sample collection and freeze the sample for molecular analysis. These findings support the ecological hypothesis where specific microbiological compositions correlate with dental caries.

## ETHICAL APPROVAL

All procedures performed in studies involving human participants were in accordance with the ethical standards of the institutional and/or national research committee and with the 1964 Helsinki declaration and its later amendments or comparable ethical standards.

## Supporting information

Table S1. Primers used for 16S rRNA sequencing of hypervariable regions V3 (amplicon 270 bp) and V6 (amplicon 191 bp).

Table S2. All phyla identified from sequencing of 16S rRNA gene sequencing in 3 saliva samples.

Table S3. Genera identified from sequencing of the 16S rRNA gene from 3 saliva samples using a cutoff of 100 reads.

Table S4. Oral health, demographics and microbial characterization of subject set. DMFT and DMFS refer to measurments of oral health on the tooth and tooth surface respectively, where upper case references permanent dentition and lower case primary dentition. DMFT_level sorts the DMFT scores into four main categories of oral health, these were used for the associations studies between microbes and oral health.

Table S5. Number of PCR‐MS Detections for Each Genera. Some subjects have multiple species from the same genera, in which case the genera will have more than one count per subject.

Table S6. Number of PCR‐MS Detections for Each Species. "/ "denotes an uncertainty among possible species assignments.

Table S7. Associations between Species and Oral Health. Relative risk (RR) was used to measure the relative risk of cavities being associate with a species being present in subjects. RR > 1 denotes higher risk of cavities with presence of species, whereas RR < 1 a lower risk of cavities with presence of species. The confindence intervals (CI) serve to estimate the true relative risk in the population (versus the sample set). The confidence level was set at 95%. NA: non‐applicable due to very low numbers.
